# Supplementary material for: Off-Label Use of Crisdesalazine (GedaCure) in Meningoencephalitis in Two Dogs
Source: Vet Sci. 2023 Jul 5;10(7):438. doi: 10.3390/vetsci10070438 (PMC10383347; doi:10.3390/vetsci10070438)
Supplement: Supplementary file 1 [file vetsci-10-00438-s001.zip › vetsci-2369065-supplementary.pdf]

Supplementary materials vetsci-2369065

Supplementary file S1. Improvement of neurological sign after adding crisdesalazine in Case 2

S1A The neurological sign (head tilt and turning) of the dog was only partially improved with cytarabine and prednisolone.

S1B After adding crisdesalazine to the ongoing treatment, neurological signs of head tilt and turning were improved

S1C After adding crisdesalazine to the ongoing treatment, the distance of linear walking was also markedly improved without increasing the dosage of immunosuppressants.
